# Neuronal and inducible nitric oxide synthase upregulation in the rat medial prefrontal cortex following acute restraint stress: A dataset

**DOI:** 10.1016/j.dib.2016.01.005

**Published:** 2016-01-13

**Authors:** Jereme G. Spiers, Hsiao-Jou Cortina Chen, Johnny K. Lee, Conrad Sernia, Nickolas A. Lavidis

**Affiliations:** School of Biomedical Sciences, The University of Queensland, St. Lucia 4072, Australia

**Keywords:** Medial prefrontal cortex, Nitric oxide synthase, Stress

## Abstract

This data article provides additional evidence on gene expression changes in the neuronal and inducible isoforms of nitric oxide synthase in the medial prefrontal cortex following acute stress. Male Wistar rats aged 6–8 weeks were exposed to control or restraint stress conditions for up to four hours in the dark cycle after which the brain was removed and the medial prefrontal cortex isolated by cryodissection. Following RNA extraction and cDNA synthesis, gene expression data were measured using quantitative real-time PCR. The mRNA levels of the neuronal and inducible nitric oxide synthase isoforms, and the inhibitory subunit of NF-κB, I kappa B alpha were determined using the ΔΔC_T_ method relative to control animals. This data article presents complementary results related to the research article entitled ‘Acute restraint stress induces specific changes in nitric oxide production and inflammatory markers in the rat hippocampus and striatum’ [1].

**Specifications table**

**Table:** 

Subject area	Neuroscience
More specific subject area	Psychoneuroendocrinology and molecular biology
Type of data	Figures
How data was acquired	Real-time PCR (QuantStudio^TM^ 6 Flex Real-Time PCR System, Applied Biosystems, Foster City, CA)
Data format	Analyzed
Experimental factors	Male Wistar rats were subjected to acute restraint stress for 0 (control), 60, 120, and 240 min in the dark-cycle. At the end of each treatment, whole brain was rapidly removed and the medial prefrontal cortex was cryo-dissected for relative gene expression analysis.
Experimental features	Total RNA was extracted from each isolated medial prefrontal cortex, reversed transcribed to cDNA, and the relative expression of neuronal nitric oxide synthase (NOS), inducible NOS, and I kappa B alpha (Nfkbia) was determined.
Data source location	Brisbane, Australia
Data accessibility	Data is within this article

## **Value of the data**

• The first genomic data demonstrating a single acute stress is capable of inducing increased neuronal NOS mRNA expression.
• The first regional observation of simultaneous increases in neuronal and inducible NOS mRNA in the medial prefrontal cortex.
• These data provide evidence for rapid inhibition of the inflammatory NF-κB pathway in the medial prefrontal cortex.

## 1. Data

**Fig. 1:**
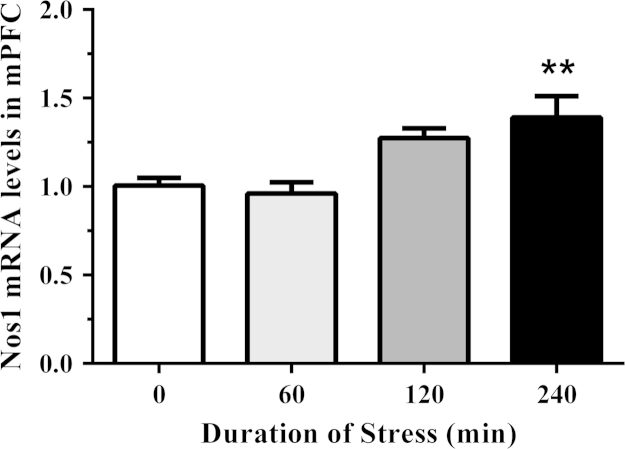
The effect of acute restraint stress on neuronal nitric oxide synthase (Nos1) mRNA expression in the medial prefrontal cortex (mPFC) of control and stressed rats (*n*=5–6 per group). The relative expression was determined in isolated mPFC collected from rats exposed to 0 (control), 60, 120, and 240 min of restraint stress. Data are expressed as mean±SEM, ⁎⁎*p*<0.01.

**Fig. 2:**
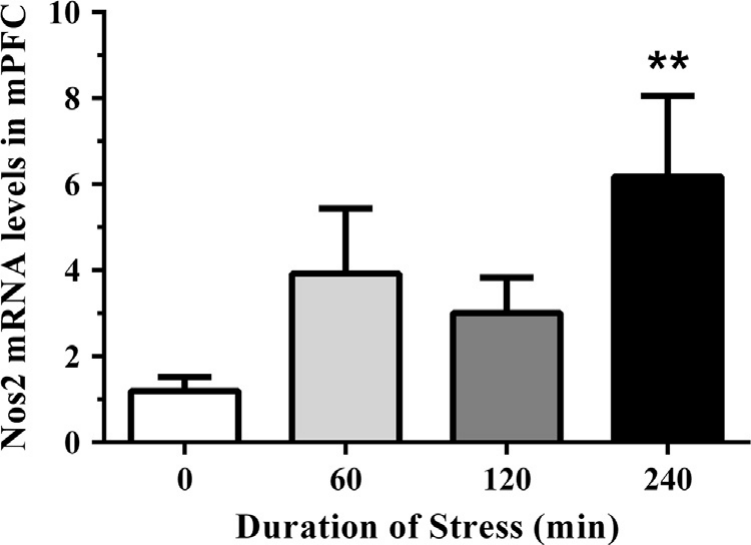
The effect of acute restraint stress on inducible nitric oxide synthase (Nos2) mRNA expression in the medial prefrontal cortex (mPFC) of control and stressed rats (*n*=5–6 per group). The relative expression was determined in isolated mPFC collected from rats exposed to 0 (control), 60, 120, and 240 min of restraint stress. Data are expressed as mean±SEM, ⁎⁎*p*<0.01.

**Fig. 3:**
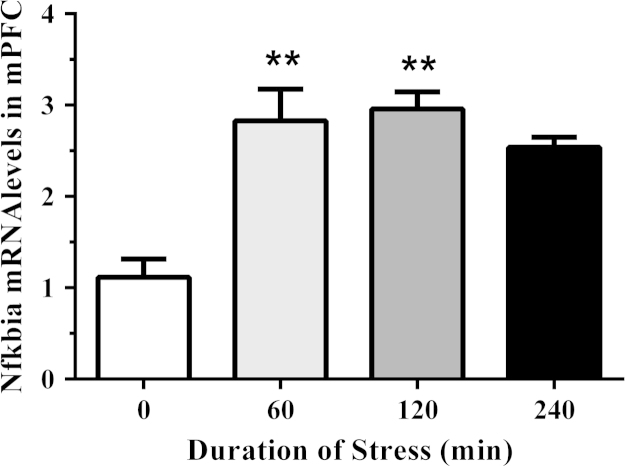
The effect of acute restraint stress on nuclear factor of kappa light polypeptide gene enhancer in B-cells inhibitor, alpha (Nfkbia) mRNA expression in the medial prefrontal cortex (mPFC) of control and stressed rats (*n*=5–6 per group). The relative expression was determined in isolated mPFC collected from rats exposed to 0 (control), 60, 120, and 240 min of restraint stress. Data are expressed as mean±SEM, ⁎⁎*p*<0.01.

We have presented genomic data from the medial prefrontal cortex in support of the research article entitled ‘Acute restraint stress induces specific changes in nitric oxide production and inflammatory markers in the rat hippocampus and striatum’ published in Free Radical Biology and Medicine [1]. The purpose of this study was to provide evidence of alterations in the nitrergic system outside of the hippocampus and striatum which have been determined previously. To achieve this, rats were exposed to control conditions, or different durations of acute stress in the dark cycle. The medial prefrontal cortex was isolated and cDNA was synthesized for relative gene expression. We have shown data for the mRNA levels of neuronal and inducible isoforms of NOS in addition to the inhibitory subunit of NF-κB (Nfkbia) relative to control animals (Fig. 1, Fig. 2, Fig. 3

## 2. Experimental design, materials and methods

### 2.1 Experimental animals

Outbred male Wistar rats (*Rattus norvegicus*) aged 6–7 weeks postnatal were housed individually in a colony room and given *ad libitum* access to standard rat chow and water. This room was on a 12 h light–dark cycle (lights off at 12.30 h) and maintained under standard laboratory conditions (22±2 °C; 55±5% humidity). Procedures performed were approved by The University of Queensland Animal Ethics Committee with AEC approval number 018/11.

### 2.2 Habituation and transportation

Animals arriving at the colony room had 7 days habituation to the new environment before experimentation. Rats were habituated to human handling daily for 10 min over 7 days. On each experimental day, rats were transported from the colony room to an experimental room within the same animal facility. Animals were acclimatized to the novel experimental room an hour before treatment with minimal sound, light, and noise.

### 2.3 Acute restraint stress

Acute restraint stress was applied for 0 (control), 60, 120, and 240 minutes (stress treatment starting at 13:30 h) within individual home cages according to Spiers and colleagues [2].

### 2.4 Treatment protocol and tissue collection

Animals were randomly allocated to 0 (control), 60, 120, or 240 min stress groups (*n*=5–6 per group). At the end of each treatment period, rats were sacrificed with sodium pentobarbital (100 mg/kg i.p. injection, Lethabarb, Virbac) and the brain removed for storage at −80 °C. Frozen brains were sectioned on a cryostat and the medial prefrontal cortex was cryo-dissected from sections according to a rat brain atlas [3]. Regionalized neural tissues were stored at −80 °C for later analysis of nNOS (Nos1), iNOS (Nos2), and Nfkbia mRNA expression.

### 2.5 mRNA expression analysis

Total RNA was extracted from each regionalized medial prefrontal cortex using the RNeasy mini kit (QIAGEN, Doncaster, Australia) and treated with deoxyribonuclease 1 (QIAGEN, Doncaster, Australia) according to the manufacturer׳s instructions. Extracted RNA was reverse transcribed into cDNA using the iScript^TM^ reverse transcription supermix (Bio-Rad Laboratories, Gladesville, Australia). The mRNA expression was determined by real-time PCR using Taqman gene expression ‘assay-on-demand^TM^’ kits (Life Technologies, Mulgrave, Australia). The primer/probe sets analyzed were FAM-labeled Nos1 (neuronal nitric oxide synthase; Rn00583793_m1), Nos2 (inducible nitric oxide synthase; Rn00561646_m1), and Nfkbia (nuclear factor of kappa light polypeptide gene enhancer in B-cells inhibitor, alpha; Rn01473657_g1). Each primer/probe was analyzed in reactions multiplexed and normalized with a VIC-labeled primer/probe assay for GAPDH (Applied Biosystems, Foster City, CA). The mRNA levels were normalized to the average of the control group using the ΔΔCT method.

### 2.6 Statistical analysis

Data were analyzed using statistical software GraphPad Prism (Version 6.0, GraphPad Software Inc., San Diego, CA, USA). One-way repeated measure ANOVAs with Dunnet׳s multiple comparisons test were used to compare normally distributed data. A non-parametric Kruskal–Wallis ANOVA with Dunn׳s multiple comparisons test was used for data with significantly different standard deviations. Results were expressed as mean±standard error of the mean (±SEM) and *p*-values less than 0.05 were considered statistically significant.
